# Advances in oxidative stress in pathogenesis of diabetic kidney disease and efficacy of TCM intervention

**DOI:** 10.1080/0886022X.2022.2146512

**Published:** 2023-02-10

**Authors:** Xiaoju Ma, Jingru Ma, Tian Leng, Zhongzhu Yuan, Tingting Hu, Qiuyan Liu, Tao Shen

**Affiliations:** a School of Basic Medicine, Chengdu University of Traditional Chinese Medicine, Chengdu, China; b School of Public Health, Chengdu University of Traditional Chinese Medicine, Chengdu, China

**Keywords:** Diabetic kidney disease (DKD), oxidative stress, pathogenesis, traditional Chinese medicine

## Abstract

Diabetic kidney disease (DKD) is a common complication of diabetes and has become the leading cause of end-stage kidney disease. The pathogenesis of DKD is complicated, and oxidative stress is considered as a core of DKD onset. High glucose can lead to increased production of reactive oxygen species (ROS) *via* the polyol, PKC, AGE/RAGE and hexosamine pathways, resulting in enhanced oxidative stress response. In this way, pathways such as PI3K/Akt, TGF-β1/p38-MAPK and NF-κB are activated, inducing endothelial cell apoptosis, inflammation, autophagy and fibrosis that cause histologic and functional abnormalities of the kidney and finally result in kidney injury. Presently, the treatment for DKD remains an unresolved issue. Traditional Chinese medicine (TCM) has unique advantages for DKD prevention and treatment attributed to its multi-target, multi-component, and multi-pathway characteristics. Numerous studies have proved that Chinese herbs (e.g., Golden Thread, Kudzuvine Root, Tripterygium glycosides, and Ginseng) and patent medicines (e.g., Shenshuaining Tablet, Compound Rhizoma Coptidis Capsule, and Zishen Tongluo Granule) are effective for DKD treatment. The present review described the role of oxidative stress in DKD pathogenesis and the effect of TCM intervention for DKD prevention and treatment, in an attempt to provide evidence for clinical practice.

## 1. Introduction

According to the *2021 International Diabetes Federation (IDF) Diabetes Atlas (10th edition)*, approximately 0.537 billion adults (20–79 years old) worldwide were affected by diabetes mellitus (DM), and the number was estimated to increase to 0.643 billion by 2030 while 0.783 billion by 2045 [1]. DM, therefore, has become a major public health issue in the world. Sustained hyperglycemia can lead to severe microvasculopathy which involves the heart, kidney, eyes, nerves, and teeth, etc. Diabetic kidney disease (DKD) is one of the common complications of DM. Clinical research demonstrated that around 20–40% of DM patients developed DKD, and in those who developed end-stage kidney disease, 80% were resulted from an interplay between hyperglycemia and hypertension. In addition, DKD was also reported as closely linked to cardiovascular disease (CVD) [2].

The main pathological changes that lead to DKD include diffuse mesangial expansion and sclerosing, alteration of the glycocalyx of endothelial cells, basement membrane thickening, and podocyte foot process effacement of glomerulus, tubular basement membrane thickening, increased apoptosis, and kidney interstitial fibrosis, which can result in persistent proteinuria and progressive reduction in glomerular filtration rate (GFR) in clinic [3]. The pathogenesis of DKD is highly complicated and remains elusive. Most scholars believe that the DKD onset is involved in multiple factors, such as insulin resistance, glycolipid metabolism disorder, hemodynamics alteration, inflammatory response, cytokine, oxidative stress, and genetic factors. Notably, oxidative stress is core to DKD onset that can be activated by the activation of the polyol pathway, accumulation of advanced glycation end-products (AGEs), and multiple cytokines and then lead to lesions in the kidney small vessels and subsequent exacerbation of kidney injury.

With the advances in purification technique, a large number of experimental studies have suggested that Chinese herbs along with their active ingredients and Chinese patent medicines can play a role in prevention and treatment of DKD with their antioxidant effects. In this review, we summarized the role of oxidative stress in DKD pathogenesis and the efficacy of TCM intervention for DKD treatment, in an attempt to instruct early interventions, help look for new therapeutic strategies and decrease the incidence and mortality of DKD.

## 2. Oxidative stress in pathogenesis of DKD

### 2.1. Overview

**Figure 1.**
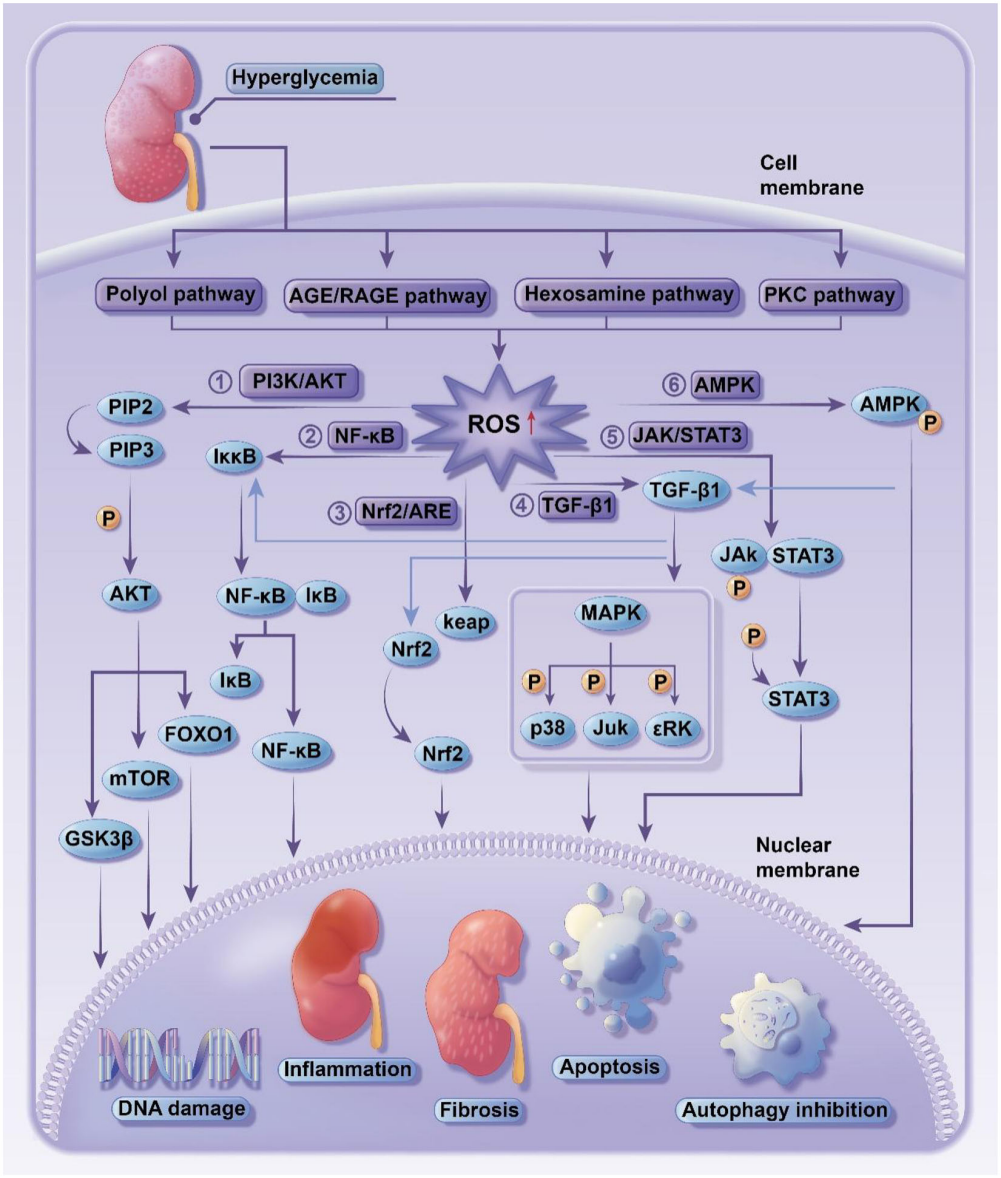
oxidative stress in pathogenesis of diabetic kidney disease.

Oxidative stress is defined as a response to oxidant-antioxidant balance disorder [4]. Upon stimulation with adverse factors from the internal and external environment, numerous reactive oxygen and nitrogen radicals are produced *in vivo* and cannot be completely scavenged with the antioxidant defense system, resulting in a series of physiological and pathological responses in cells and tissues. In addition, the interplay between multiple factors, such as glycolipid metabolism disorder and hemodynamics alteration, activates pathways like polyol and hexosamine pathways to induce substantial ROS production, leading to oxidant-antioxidant balance disorder in the kidney and inducing oxidative stress responses. As a consequence, the downstream cellular signaling pathways are activated, which causes inflammation, autophagy, and fibrosis, etc., accelerating the pathological changes and functional abnormality that led to DKD (Figure 1).

### 2.2. Upstream pathways that activate oxidative stress response

#### 2.2.1. Polyol pathway

Polyol pathway is one of the metabolic pathways [5] from glucose reduced to sorbitol under the actions of aldose reductase (AR) and nicotinamide adenine dinucleotide phosphate (NADPH) and subsequently oxidized to fructose in presence of sorbitol dehydrogenase (SDH) and nicotinamide adenine dinucleotide (NAD). In states of hyperglycemia, AR is activated, leading to increased production of sorbitol. Due to the consistent SDH activity, the produced sorbitols are accumulated in cells, which causes an increase in cell membrane permeability, resulting in exudation of intracellular matters such as inositol and reduced glutathione (GSH) and eventually oxidative stress response [6]. During this metabolic process, the activation of AR is dependent on NADPH, while the metabolism of excessive glucose consumes large amounts of NADPH, leading to reduced GSH production [7] and ROS scavenging capacity, eventually resulting in redox balance disorder *in vivo*. In the meantime, SDH-induced nicotinamide adenine dinucleotide (NADH) increases and then is oxidized to superoxides and other ROS under respiratory chain functions in mitochondria [8]. The accumulation of end product fructose can also induce oxidative stress and subsequent oxidative damage to tissue. Research revealed that AR inhibitor could reduce oxidative stress in the kidney to protect kidney function, which suggested that polyol induces DKD onset through inducing oxidative stress responses [9].

#### 2.2.2. Ages/RAGE pathway

AGEs are highly active, irreversible end products of non-enzymatic reactions between glucuronyl and free amino groups such as lipid and protein [10]. Sustained hyperglycemia stimulation accelerates AGEs production, while the excessive AGEs directly increase ROS production. Notably, the generated ROS feeds back to stimulate AGEs production, exacerbating oxidative stress and resulting in renal tissue damage.

A large body of research suggested that receptor for AGEs (RAGE) is increasingly expressed by glomerular epithelial cells, mesangial cells, endothelial cells, and podocytes upon hyperglycemia stimulation. The RAGE binding to AGEs activates NADPH oxidase to increase ROS production in endothelial cells, which disturbed molecular conformation and altered enzyme activity, inducing oxidative stress responses. As a consequence, the activated oxidative stress mediates the downstream signaling pathways (e.g., NF-κB, TNF-β, JNK, and p38-MAPK [11]) to increase the release of adhesion molecules, vascular endothelial factors and inflammatory factors [12], leading to multiple mechanisms that contribute to renal injury, such as renal interstitial fibrosis and mesangial expansion.

#### 2.2.3. Hexosamine pathway

In the glycolysis pathway, approximately 2–5% glucose-6-phosphate (G6P) is converted to fructose-6-phosphate (F6P) and then enters the hexosamine pathway [13]. At states of sustained hyperglycemia, excessive F6P is converted to glucosamine-6-phosphate (GlcN6P) under the catalysis of glutamine fructose-6-phosphate aminotransferase (GFAT), followed by generation of uridine diphosphate N-acetylglucosamine (UDP-GlcNAc) with the action of related enzymes. The UDP-GlcNAc is then used as substrate for O-linked N-acetylglucosamine (O-GlcNAc) glycosylation under the catalysis of O-GlcNAc transferase. It was reported that hexosamine can induce endoplasmic reticulum (ER) stress in endothelial cells and macrophages, leading to increased oxidative stress responses. Another study revealed that overexpression of GFAT increases NF-κB promoter activity and TNF-α expression in mesangial cells and stimulates the production of TGF-β1 and PAI-1, inducing inflammatory response, extracellular matrix (ECM) accumulation and diabetic glomerulosclerosis [14].

A recent study showed a close link between O-GlcNAc glycosylation and DKD. The intracellular O-GlcNAc modulates the mitochondrial stress, impairing endothelial nitric oxide synthase (eNOS) activity while increasing uperoxide anion generation to regulate ROS production. In the meantime, O-GlcNAc modifies specific proteins to stimulate the production of large amounts of AGEs, subsequently inducing a series of pathological changes (e.g., apoptosis, mesangial cell proliferation, and fibrosis) that lead to progression of DKD [15].

#### 2.2.4. Pkc pathway

The protein kinase (PKC) pathway is critical in the oxidative stress-induced DKD occurrence and development. Under normal circumstances, the PKC in renal tissue is in an inactivated state. While upon hyperglycemia, it will be activated given the significant increase in intracellular diacylglycerol content [16]. Additionally, PKC can also be activated indirectly *via* AGE/RAGE and polyol pathways [17]. Activated PKC enhances NADPH oxidase activity and promotes the endothelial and mesangial cells to produce ROS, resulting in damage to renal tissue cells from oxidative stress [18].

Moreover, the activated PKC can also change renal hemodynamics by increasing prostaglandin (PG) and nitric oxide (NO) [19]; elevate the expression of vascular endothelial growth factor (VEGF) and vascular permeability factor (VPF) and decrease glomerular Na+-K+-ATPase activity [20]; increase TGF-β expression to cause renal basement membrane thickening [21]. Besides, the ROS produced following PKC pathway activation can induce apoptosis in cells, exacerbating kidney injury [22].

### 2.3. Downstream signaling of oxidative stress

#### 2.3.1. Pi3k/Akt pathway

Phosphoinositide 3-kinase (PI3K)/protein kinase B (Akt) pathway mainly acts in regulation of cell proliferation, differentiation and apoptosis. Upon an extracellular stimulus, PI3K phosphorylates phosphatidylinositol 4,5-bisphosphate (PIP2) to generate phosphatidylinositol-3,4,5-triphosphate (PIP3). The generated PIP3 further induces Akt plasma membrane translocation and activates Akt under the catalysis of phosphoinositide-dependent kinase-1 (PDK1) [23]. The activated Akt then plays a role *via* regulating downstream signaling molecules such as glycogen synthase kinase (GSK) 3β, mammalian target of rapamycin (mTOR) and forkhead box protein O1 (FoxO1) [24].

##### 2.3.1.1. Pi3k/Akt/GSK3β signaling pathway

GSK3β is the downstream target of Akt. Activated Akt phosphorylates GSK3β and blocks its activity to modulate various cellular functions. Previous research found that sustained high glucose levels resulted in suppressed PI3K/Akt signaling, leading to decreased GSK3β activity, reduced Bcl-2 but elevated Bax, which induced apoptotic damage to renal cells [25]. Animal experiment noted that phosphorylated GSK3β augmented NF-κB function to increase inflammation in DKD mice [26]. In addition, another study showed that reduced GSK3β activity increased β-catenin and slowed the degradation of snali, leading to increased trans-differentiation of tubular epithelial cells and promoting renal interstitial fibrosis in diabetic rats [27].

##### 2.3.1.2. Pi3k/Akt/mTOR signaling pathway

mTOR has been the focus of intense research, and it is tightly linked to the Akt-mediated signaling pathways. Activated Akt phosphorylates and activates mTOR and downstream signaling pathways to participate in proliferation, apoptosis and glucose metabolism in cells. Lu Q et al. [28] found that high glucose levels induced tubular epithelial cells NRK-52E to produce ROS, stimulating TGF-β1 generation and Akt activation, while the activated Akt phosphorylated mTOR to play a role in epithelial-mesenchymal transition (EMT) in NRK-52E cells, exacerbating diabetic kidney fibrosis. Another study reported that high glucose levels also activated the mTOR signaling to reduce LC3II/LC3I ratio and Beclin level, thereby decreasing autophagy to make effects on DKD progression [29].

##### 2.3.1.3. Pi3k/Akt/FoxO1 signaling pathway

FoxO1 is a class of highly conserved, ubiquitously expressed transcription factors that has implications for processes such as oxidative stress, inflammation, autophagy and apoptosis upon high glucose stimulation. PI3K/Akt signaling phosphorylates FoxO1 and deactivates it *via* inducing its nuclear translocation, the process of which is significant for the pathogenesis of diabetic kidney injury [30]. FoxO1 in renal tissue can alleviate the impaired glomerular filtration barrier and apoptosis resulted from the detachment of renal podocytes from the basement membrane because of abnormal glucose metabolism [31]. Early elevation of glucose can stimulate the TGF-β/Smad pathway to prevent the occurrence of kidney fibrosis in DKD [32]. The study of Ma et al. [33] also found that increased phosphorylation of FoxO1 decreased FoxO1 activity and autophagy in DKD animal models, resulting in exacerbation of kidney injury.

#### 2.3.2. Nf-кB signaling pathway

In normal cases, NF-кB is ubiquitously present in various tissue cells in an inactive state. In response to stimulation with activators (e.g., proinflammatory cytokine, Toll-like receptor, p38-MAPK, HO-1, and ROS), NF-κB dimers will be released to nucleus to regulate target gene expression, inducing the body’s immune and inflammatory responses [34]. Multiple studies have proved that DKD development is associated with NF-κB hyper-activation. For example, the produced ROS and inflammatory factors (e.g., TNF-α and IL-6) under high glucose stimulation activated the NF-κB signaling, while the activated NF-κB then promoted the transcription and translation of proinflammatory cytokines, chemokines, adhesion molecules, and TGF-β1, etc., leading to apoptosis and necrosis in cells and tissue fibrosis, which accelerated the development of DKD [35]. Hofmann et al. [36] found that NF-κB expression in DKD patients was significantly increased in comparison to that in non-diabetic patients, and it was positively associated with the degree of proteinuria in this population.

Collectively, suppression of the NF-κB signaling pathway activation may be a direction for research in prevention and treatment of DKD.

#### 2.3.3. Nrf2/ARE signaling pathway

Nuclear factor erythroid 2-related factor 2 (Nrf2) is a transcription factor important in body’s resistance to oxidative stress. It is involved in regulation of anti-oxidant, anti-inflammatory, anti-apoptosis, and anti-fibrostic processes and is closely associated with the degree of DKD injury. Under normal physiological conditions, Nrf2 exists in cytoplasm in an inactive state as a compound with its inhibitor Kelch-like ECH-associated protein 1 (KEAP1). High glucose-induced oxidative stress dissociates Nrf2 from the compound and activates it to induce nuclear translocation. The activated Nrf2 binds with the antioxidant response elements (AREs) in nucleus to activate the expression of downstream antioxidant factors, playing a protective role in cells and then slowing the development of kidney injury in DKD [37]. An animal experiment demonstrated that Nrf2-/- mice suffered from severer kidney injury than wild mice, which also proved the protective role of Nrf2 in kidney diseases [38]. Combining the findings, Nrf2 could be employed as a key target to prevent DKD and preserve renal function.

Animal experimental data revealed that inhibiting the Nrf2-mediated activities of antioxidant enzymes, including superoxide dismutase (SOD), catalase (CAT) and glutathione peroxidase (GSH-Px), exacerbated the kidney injury in mice with spontaneous DKD [39]. Another study noted that inhibition of the Nrf2 signaling while activating the TGF-β1-stimulated fibronectin expression and smad2/3 phosphorylation slowed down the occurrence of renal fibrosis in rats with streptozotocin (STZ) -induced diabetes [40]. Additionally, up-regulation of Nrf2 down-regulated TNF-α, IL-6, Bax and p38 levels, suppressed NF-κB activation and increased Bcl-2 expression, showing anti-inflammatory and anti-apoptotic effects, conducive to preventing the STZ-induced DKD [41].

#### 2.3.4. Tgf-β1 signaling pathway

During the development of DKD, oxidative stress activates the TGF-β1 signaling pathway to induce expression of intracellular signals (protein kinase or cytokine), which promotes ECM accumulation and induces EMT, resulting in renal interstitial fibrosis and glomerulosclerosis [42]. A meta-analysis of randomized controlled trials (RCTs) demonstrated that serum TGF-β1 could be employed as a biomarker for early warning of fibrosis in DKD [43]. In addition, it was proven that TGF-β1 regulators, such as Resveratrol, Salvia Root, Taxol and Calcitriol, could improve the fibrosis in DKD *via* inhibiting the TGF-β1 signaling pathway [44].

It has been established that the profibrotic effect of TGF-β1 is regulated by multiple signaling pathways, among which the mitogen-activated protein kinase (MAPK) signaling pathway has received increasing attention. The MAPK family contains three important members: p38-MAPK, JNK and ERK, which are intimately associated with the development of DKD. Notably, p38-MAPK shows the strongest relationship with DKD. In response to high glucose, pro-inflammatory factor stimulation and oxidative stress, activated p38-MAPK targets and acts on TGF-β1, promoting the phosphorylation of Smad2/3 and increasing the expression of fibronectin, collagen I and IV (Col-I/IV) [45], thereby playing a role in renal fibrosis. In addition, p38-MAPK with enhanced activity in turn exacerbates the body’s oxidative stress, resulting in increased oxygen radicals [46].

ERK as another member of the MAPK family is also associated with the TGF-β1 signaling. In animal models of kidney fibrosis from many causes, the TGF-β1/ERK pathway was activated, while suppression of the pathway improved the kidney fibrosis [47]. Another study noted that activated ERK signaling induced the transcriptional regulation of hypoxia inducible factor-1α (HIF-1α) to regulate the levels of fibronectin, Col-I and connective tissue growth factors (CTGF) [48], thereby playing a role in multiple pathological processes of DKD such as proliferation, hypertrophy and increased ECM in glomerular mesangial cells.

Therefore, TGF-β1 is a key factor that induces fibrosis in DKD.

#### 2.3.5. Jak2/STAT3 signaling pathway

The Janus kinase (JAK)/signal transducer and activator of transcription (STAT) signaling pathway is activated in DKD, while inhibition of this pathway can slow down the progression of this disease [49]. Experiment in DKD rats revealed increased phosphorylation of JAK and STAT3 in renal tissue, leading to up-regulated Bax while down-regulated Bcl-2, which enhanced apoptosis in renal cells [50]. The JAK2/STAT3 pathway can also activate macrophages in the kidney to promote the release of inflammatory factors and the production of ROS, exacerbating kidney injury [51]. It was reported that Berberine alleviated the high glucose-induced EMT and apoptosis in renal podocytes by suppressing the JAK2/STAT3 signaling pathway [52], thereby slowing down the progression of DKD.

Collectively, the JAK2/STAT3 signaling pathway mainly participants in the regulation of immune response, inflammation, oxidative stress and cell apoptosis during the course of DKD.

#### 2.3.6. Ampk signaling pathway

Adenosine monophosphate-activated protein kinase (AMPK) is a sensor of cellular energy, and it has gained intensive attention given its role as a therapeutic target for obesity, diabetes, and metabolic syndrome-related diseases. Clinical research revealed that phosphorylated AMPK (p-AMPK) protein expression significantly decreased in renal tissue of DKD patients, and AMPK activator improved the clinical symptoms and pathological changes and reduced 24-h urinary proteins (UP) in these patients [53]. Additionally, p-AMPK in human glomerular cells upon high glucose reduced the expression of nicotinamide adenine dinucleotide phosphate oxidase (NOX) 4 and TGF-β1 and subsequent ECM accumulation [54]. AMPK also serves as a sensor of intracellular ROS. Activated AMPK up-regulates the expression of SODs to reduce superoxide generation, and it phosphorylates and activates FOXO to exert antioxidant effects. It was proven that the levels of AMPK, p-AMPK and SIRT1 were remarkably decreased in both mouse models of STZ-induced DKD and podocytes exposed to high glucose, activating oxidative stress and then leading to pyroptosis. In addition, inhibiting this process suppressed the injury in DKD [55]. Upon high glucose, AMPK phosphorylation is inhibited, leading to increased PPAR-α expression, lipid accumulation, apoptosis, and expression of pro-inflammatory and profibrotic gene expression [56]. In the *in vivo*/vitro experiments performed by Han YC et al. [57], AMPK activator promoted mitophagy *via* activating the p-AMPK-pink1-pinkin pathway, thereby improving the oxidative stress and interstitial fibrosis in the kidney.

## 3. Advances in Chinese medicine in treatment of DKD

Current treatment for DKD mainly aims at controlling body weight, lowering blood glucose and pressure (antihypertensive drugs such as ACEI/ARB), and inhibiting the renin-angiotensin-aldosterone system (RAAS) system. However, such treatment fails to stop proteinuria progression and the persistent changes of organ functions [58]. Clinical research and *in vivo*/vitro experiments have revealed that Chinese herbal medicines (e.g., Milkvetch Root, Kudzuvine Root), their bioactive ingredients (e.g., ristocetin, berberine), and Chinese patent medicines (e.g., Yishen Tongluo Granule) can slow down DKD progression *via* multiple signaling pathways and mechanisms (e.g., anti-oxidant, anti-inflammatory, anti-fibrotic, and podocyte-protective), showing certain advantages with respect to clinical efficacy for DKD treatment [59]. Chinese medicines have great potential for therapeutic applications for DKD treatment. The following part reviewed the recent advances in the representative Chinese herbal medicines, active ingredients and patent medicines with therapeutic efficacy for DKD.

### 3.1. Chinese herbal medicines and active ingredients

It has been proven that Chinese herbal medicines and their bioactive ingredients, such as polyphenols (e.g., breviscapine, puerarin, resveratrol, safflower yellow, silymarin, curcumin, icariin, and tanshinone), polysaccharides (e.g., lycium barbarum polysaccharides, astragalus polysaccharides, ramulus mori polysaccharides, and Chinese Yam polysaccharides), alkaloids (e.g., berberine, tripterygium glycosides, ligustrazine, and emodin), and saponins (e.g., astragaloside and ginsenoside), can improve glucose metabolism and reduce renal pathological changes *via* multiple anti-inflammatory and anti-fibrotic pathways involved in oxidative stress, playing a nephroprotective role. See Table 1 for more details.

**Table 1.** Clinical efficacy and mechanism of action of Chinese herbs and active ingredients for DKD treatment.

Categories	Component	Result	Pathway
Tripterygium	Tripterygium glycosides	Reduce 24-h UP, UAER and urinary β2-microglobulin	Up-regulate autophagy through mTOR/Twist1, inhibit TLR4/NF-κB to decrease EMT and apoptosis/PI3K/Akt, immune-inflammatory [60–63]
	Triptolide		
Polyphenols	Puerarin	Reduce UAER and SCr	RAGE pathway, regulate SIRT1 to induce autophagy and reduce PKC activity [65–68]
	Breviscapinun	Reduce 24-h UP, SCr, BUN, and elevate TC and TG to decrease dyslipidemia	Regulate the Nrf2 /HO-1, TGF-β1, and PKC pathways [69–71]
	Resveratrol	Improve UACR, and increase production of antioxidant enzymes	Inhibit caspase 3, TGF-β/Smad and ERK1/2 to reduce early glomerulosclerosis [73–76]
	Icariin	Blood lipid-lowering, anti-aging, and anti-inflammation	TLR4/NF-κB, Keap1/Nrf2, Col-IV/TGF-β1, miR-192-5p/GLR-1R [77–80]
	Silymarin	Reduce UACR, urinary TNF-α, and MDA	Protect against kidney fibrosis *via* the JAK/STAT3/SOCS1 and TGF-β1/Smad pathways [82–84]
	Curcumin	Anti-oxidization and anti-inflammation	Regulate Nrf2 to inhibit NF-κB, TGF-β1, CTGF, fibronectin and Col IV [85–88]
	Sal*via*nolic acid	Reduce blood glucose	Suppress TNF-α and IL-6 [89]
	Safflower amine	Inhibit platelet/neutrophil adhesion, decrease fibrosis [90]	
Polysaccharides	Lycium barbarum polysaccharides	Enhance activity of antioxidant enzymes and scavenge of oxygen radicals	Decrease ERK 1/2 activation *via* the PKC pathway [91–93]
	Ramulus mori polysaccharides	Dispel wind and activate collaterals, ease joint movement, dry dampness and alle*via*te water retention, regulate glycolipid metabolism, and improve the body’s immune function	Up-regulate the expression of SIRT1 and FoxO1 proteins and decrease NF-KB to resist oxidative stress [94]
	Chinese yam polysaccharides	Elevate insulin level, decrease glucagon level and FBG	Enhance multiple anti-oxidative stress pathways including the PI3K [95]
Alkaloids	Berberine	Reduce UER, FBG, HbA1c and GPx	Regulate the expression of GRKs in the G protein-AC-cAMP signaling pathway [99–104]
	Ligustrazine	Reduce 24-h UP, blood glucose, and BUN	Anti-oxidative stress, anti-inflammation, and anti-fibrosis [106–107]
Saponins	Astragalosides	Alle*via*te albuminuria and glomerulosclerosis, and simultaneously inhibit podocyte apoptosis and recover impaired autophagy	PI3K/Akt/FoxO1, AMPK and SIRT/NF-κB signaling pathways [108–116]
	Paeoniflorin	Reduce UAER	Alle*via*te oxidization and inflammation through the TLR2/4 and JAK2/STAT3 pathways, up-regulate p-mTOR level to promote autophagy [117–119]
	Ginsenoside	Reduce blood glucose and lipid levels, improve oxidative stress	Akt/GSK-3β, PI3K/Akt, TGF-β1/Smad [121–126]
	Notoginsenoside	Reduce FBG, Ccr, UAlb and kidney index	Enhance BMP-7 and Smad7 *via* inhibiting VEGF and TGF- β1 [127]
Other	**Emodin**	Reduce 24-h UP	Regulate the AMPK/mTOR-mediated autophagy and activate the PI3K/Akt/GSK-3β pathway to inhibit inflammation [128–131]

#### 3.1.1. Tripterygium

Tripterygium and its extracts (e.g., triptolide and tripterygium glycosides) have been recognized as important therapeutic agents for clinical treatment of DKD with proved efficacy. A meta-analysis in 2,764 DKD patients from 31 articles revealed that Tripterygium Glycosides Tablet administration for 3 months significantly reduced the levels of 24-h uridine triphosphate (UTP) and serum creatinine (SCr) in DKD patients, and there was a low incidence of adverse events [60]. Another study found that tripterygium glycosides up-regulated the autophagy level and reduced the EMT and apoptosis in DKD rats through the mTOR/Twist1 signaling pathway [61]. Moreover, it was also noted that tripterygium glycosides inhibited tubular fibrosis *via* inhibiting TLR4/NF-κB to improve EMT or prevented DKD development through suppressing the immune-inflammation system *via* the PI3K/Akt pathway [62].

Triptolide is another important active ingredient of Tripterygium. Research revealed that triptolide could activate SOD to inhibit renal inflammation, macrophage infiltration and GSK3β phosphorylation, thereby alleviating proteinuria and podocyte injury in DKD rats [63].

#### 3.1.2. Polyphenols

Polyphenols, mainly including flavonoids, tannins and phenolic acids, are natural compounds with multiple phenol groups. They have a wide range of clinical applications due to their strong antioxidant capability and proved efficacy in reducing blood glucose and urinary albumin and increasing GFR.

Puerarin, an extract of Kudzuvine Root, is mainly composed of flavonoid C-glycoside [64]. Clinical research demonstrated that puerarin injection led to remarkable reductions in urinary albumin excretion rate (UAER) and SCr, showing good therapeutic efficacy [65]. While in STZ-induced DKD animal model and high glucose-treated podocytes, puerarin protected the kidney *via* activating SIRT1 to induce autophagy and through its antioxidant effect [66]. The study of Yuan et al. [67] reported that puerarin inhibited AGEs formation to block AGEs binding to RAGE, thereby improving the pathological changes that lead to DKD. Furthermore, it was also noted that puerarin could decrease PKC activity and down-regulate expression of c-Fos, c-Jun and Col-IV to reduce ECM accumulation [68].

Breviscapinun is the purified flavonoid extracted from Erigeron Breviscapus and mainly comprising breviscapinun-7-glucuronide (90%) and apigenin 7-O-glucoside (4%). In a meta-analysis in 2,320 DKD patients, breviscapinun injection combined with routine treatment contributed to significant reductions in patient 24-h UTP, SCr, blood urea nitrogen (BUN), UAER and albumin (ALB), suggesting advantages of breviscapinun injection in efficacy for DKD [69]. Xu et al. [70] found that breviscapinun was capable of inhibiting the oxidative stress, PKC activity increase and TGF-β1 overexpression in DKD rats, conducive to decreasing the pathological kidney injuries in DKD. While in the study of Jiang et al. [71], breviscapinun was found to exert anti-fibrotic effects in the kidney through reducing the phosphorylation levels of PKCβII, Akt, JNK1/2 and p38.

Resveratrol is a representative polyphenol that exists in Chinese herbal medicines such as Giant Knotweed Rhizome and Cassia Tora as a strong antioxidant. Clinical trial revealed that the combination of resveratrol (500 mg/d) with Losartan (12.5 mg/d) reduced the urinary albumin-creatinine ratio (UACR) (–46.4 mg/g, 95% CI: −64.5 to −28.3 vs 29.9 mg/g, 95% CI: 4.9 to 54.9; *p* < 0.001) and increased the activity of antioxidant enzymes [72]. A meta-analysis involving 36 animal studies demon strated that resveratrol exerted its antioxidant effects by means of reducing malondialdehyde (MDA) level and restoring activities of SOD, CAT, GSH and GPx [73]. Mechanistic studies showed that resveratrol played its therapeutic role in DKD treatment under multiple mechanisms: (1) ameliorate oxidative stress and reduce apoptosis *via* up-regulating AMPK expression and activity [74]; (2) regulate autophagy through overexpressing miR-18a-5p to suppress Caspase 3 [75]; (3) alleviate early-stage glomerulosclerosis *via* inhibiting TGF-β/Smad and ERK1/2 [76].

Icariin is a type of flavonoid derived from the dry cauline leaf of Epimedium Herb, with blood lipid-lowering, anti-aging, and anti-inflammatory pharmacological effects. It is also reported to have a protective role against DKD. Qi MY et al. [77] found that icariin suppressed the TLR4/NF-κB signaling pathway, reduced inflammatory response, and increased the activity of SOD, CAT and GSH-Px. The *in vivo*/vitro experiments performed by Wang et al. [78] showed that icariin decreased the level of superoxide anion *via* GPER-mediated Keap1 degradation and Nrf2 activation, enhancing the activity of antioxidant enzymes while reducing apoptosis and fibronectin formation, which subsequently alleviated the kidney injury in DKD. In addition, multiple studies have proved that icariin can delay the progression of DKD with its anti-fibrotic effects. For instance, icariin could down-regulate Col-IV and TGF-β1 expression [79] or enhance autophagy through the miR-192-5p/GLR-1R pathway to reduce tubulointerstitial fibrosis [80].

Silymarin is a natural bioactive substance extracted from the dry fruit of Silybum Marianum. In a RCT with 60 DKD patients, patients administrated with 12-week renin-angiotensin system (RAS) inhibitors plus capsules of silymarin (140 mg/d, tid) had significantly reduced levels of UACR, urinary TNF-α and MDA in comparison to patients administrated with RAS inhibitors alone [81]. Silymarin also has antioxidant effects. Khazim et al. [82] found that silymarin reduced NADPH enzyme, Nox4 activity, and superoxide production and apoptosis in cells, protecting podocyte from damages caused by high glucose. In addition, the study of Chen et al. [83] showed that silymarin was capable of reducing the IL-6 and ICAM-1-induced inflammatory response *via* inhibiting the JAK/STAT3/SOCS1 pathway, and it also played an anti-fibrotic role through suppressing the TGF-β1/Smad signaling pathway, eventually improving the STZ-induced kidney injury in diabetic rats. Liu et al. [84] also noted that silymarin activated the Akt signaling pathway and reduce the levels of p-GSK-3β and Bax to prevent kidney injury and alleviate oxidative stress in db/db mice. Silymarin, therefore, is promising to be applied as a new agent for DKD treatment.

Curcumin is the active ingredient extracted from the rhizomes of Curcuma longa. Growing studies have proved the protective effect of curcumin against DKD, which is achieved by its anti-oxidant and anti-inflammatory effects. For example, ALTamimi JZ et al. [85] established an animal model of DKD and found that curcumin reduced kidney injury *via* activating Nrf2 while decreasing NF-κB to inhibit NADPH oxidase and down-regulate the PKCβII/p66Shc axis. Soetikno et al. [86] also identified that curcumin modulated PKC activator-mediated phosphorylate ERK1/2 to reduce oxidative stress, and in the meantime, decreased the expression levels of TGF-β1, CTGF, fibronectin and Col-IV. The study of Tu et al. [87] reported that curcumin administration led to reduced levels of blood glucose, SCr, BUN, and UP, up-regulated expression of E-cadherin and LC3, and suppressed PI3K/Akt/mTOR pathway in rats with STZ-induced DKD, inducing autophagy while decreasing EMT. Moreover, Tikoo et al. [88] noted that the protective effect of curcumin against DKD might be attributed to its actions on post-translational modification of histone H3 and p38-MAPK expression.

There are some other polyphenols with therapeutic efficacy against DKD. Salvianolic acids, for example, could inhibit TNF-α and IL-6 to improve the STZ-induced glucose elevation in rats, protecting DKD rats from oxidative stress injury [89]. Safflower yellow is the safflower amine component extracted from Safflower carthamus with characteristics of inhibiting platelet/neutrophil adhesion, vascular endothelial injury and smooth muscle hyperplasia, and improving development of fibrosis in DKD [90].

#### 3.1.3. Polysaccharides

Lycium barbarum polysaccharides (LBP) are main extracts of the fruit of wolfberry, and they have been intensively applied in China for their immunomodulatory, liver protective, anti-oxidant, and anti-tumor effects. Both domestic and abroad studies have proved that LBP have protective effects in DKD. For instance, Du et al. [91] identified that LBP decreased TLR4, Myd88 and NF-κB expression to reduce the release of inflammatory factors such as IL-2, IL-6 and TNF-α and enhance the activity of serum SOD and GSH-Px, thereby exerting anti-inflammatory and anti-oxidant effects. Other studies reported that LBP could reduce mesangial cell proliferation through the PKC and ERK1/2 pathways [92]. In addition, it could also inhibit the binding between endothelin and its receptor, resulting in decrease in UAER level and subsequent improvement of kidney function [93].

Ramulus mori polysaccharides (RMP) are one of the main active ingredients of mulberry branch that have functions of drying dampness and alleviating water retention, lowering blood glucose and lipid, and improving immunity. Research revealed that RMP could improve the oxidative stress-induced alteration of renal function *via* decreasing the content of MDA and ROS, up-regulating the expression of SIRT1 and FoxO1 proteins, and reducing the levels of inflammatory factor NF-κB [94].

Chinese yam polysaccharides (CYPS) were reported to alleviate the kidney injury in DKD through enhancing the PI3K signaling pathway *via* actions on the expression of IRS-1 and PI3K [95]. Other polysaccharides, such as the polysaccharides from cyclocarya paliurus, alga and okra also had a protective role in DKD rats *via* exerting their anti-oxidant effect [96].

#### 3.1.4. Alkaloids

Berberine, the main bioactive extract of Golden Thread and Amur Cork-Tree, is a benzylisoquinoline alkaloid structurally with heteropentacyclic groups [97]. It has broad clinical applications owing to its effects to regulate glycolipid metabolism and protect the kidney *via* anti-inflammatory and anti-oxidant mechanisms [98]. A previous RCT revealed that berberine led to reductions in 24-h UP, UAER, fasting blood glucose (FBG), glycated hemoglobin (Hb A1c), total cholesterol (TC), C-reactive protein (CRP), and GPx [99]. In addition, an animal experiment found that berberine increased the glucose uptake, lipid oxidization, and insulin sensitivity in diabetic fatty rats [100]. Mechanistic studies showed that berberine regulated the G protein-coupled receptor kinase (GRK) of the G protein-AC-cAMP signaling pathway to increase the cAMP level in STZ-induced diabetic rats [101]; and it reduced interstitial fibrosis in DKD by inhibiting the EMT in tubular epithelial cells *via* activating Nrf2 while decreasing TGF-β/Smad expression [102]. Moreover, *in vitro* studies reported that berberine activated PGC-1α to restore mitochondrial function, blocking the excessive production of ROS [103]; and it regulated autophagy *via* activating the AMPK signaling or inhibited TLR4/NF-κB to improve the high glucose-induced podocyte apoptosis [104].

Ligustrazine is an alkaloid monomer derived from the rhizome of Sichuan Lovage Rhizome. A huge number of clinical and animal studies have demonstrated the preventive effect of ligustrazine on DKD. For instance, a meta-analysis of 25 RCTs involving 1,645 DKD patients (ligustrazine group, *n* = 858; control group, *n* = 787) revealed that ligustrazine injection reduced BUN, SCr, 24-h UP, and UAER in these patients, exhibiting certain clinical efficacy [105]. Similarly, in another meta-analysis, the combination of Salvia Root and ligustrazine also decreased the body’s inflammatory response, and it was safe and effective in early DKD treatment as an adjuvant therapy [106]. Furthermore, there was a meta-analysis in animal experiments, which showed that ligustrazine (150 mg/kg) improved the glomerular and tubular pathological changes and the indices of renal function. It was believed that such effects were attributed to the anti-oxidant, anti-apoptotic and anti-inflammatory activities of ligustrazine as well as its effects to reduce renal fibrosis, inhibit mesangial cell proliferation and promote autophagy [107].

#### 3.1.5. Glycosides

Astragaloside is the main bioactive component of Milkvetch Root (astragalus) that has a nephroprotective function. Milkvetch Root is clinically applied for treatment of CVD, kidney and liver diseases, etc., especially common in DKD treatment [108]. Clinical research indicated that astragalus injection reduced the BUN, SCr, UP, and increased the creatinine clearance (Ccr) and se rum albumin in DKD patients [109]. In addition, both astragalus injections with and without ACEI/ARB had significant nephroprotective effect in DKD patients, presenting as remarkably decreasing albuminuria and slowing down progression of glomerulosclerosis. In the meantime, there were no severe adverse events during the treatment as supported by a large number of samples, showing a safety profile [110]. Furthermore, in a meta-analysis that included 424 animals from 24 studies, astragalosides were proven to exert effects in reducing ER stress, inhibiting mitochondrial division and increasing autophagy activity *via* anti-fibrotic, anti-oxidant, and anti-apoptotic mechanisms [111]. More specifically, astragalosides inhibited the apoptosis in tubular epithelial cells and reduced TGF-β1 expression [112]; regulated Nrf2 and PINK1 to improve the podocyte injury and mitochondrial function in diabetic rats [113]; suppressed the PI3K/Akt/FoxO1 signaling or activated the AMPK to mediate the autophagy activity of renal tissue cells, alleviate albuminuria and glomerulosclerosis, and inhibit podocyte apoptosis [114]; modulated the SIRT1/NF-κB signaling and decreased the excessive ECM production to inhibit high glucose-induced mesangial fibrosis [115]; and inhibited the NLRP3-mediated inflammatory response in turn to improve renal function and podocyte injury [116].

Paeoniflorin is the purified bioactive component of Chinese herbaceous peony. It has been applied for treatment of chronic kidney diseases due to it biological effects such as anti-oxidization and anti-inflammation, and it has also shown a protective role in DKD. Animal experiments demonstrated that paeoniflorin inhibited the TLR2/4 signaling pathway [117], or suppressed the JAK2/STAT3 pathway to block macrophage activation and subsequently reduce kidney injury [118]. *In vitro* experiments proved that paeoniflorin decreased AGEs generation, increased activity of GPx and catalase, reduced the levels of IL-6 and MCP-1, thereby alleviating oxidative damage and inflammation in cells [119]. In addition, it could also up-regulate p-mTOR level to promote autophagy. Combining the studies, paeoniflorin has the potential as an agent for prevention and treatment of DKD [120].

Ginsenoside, belonging to Araliaceae ginseng species, is mainly composed of Rh2, Rg1, Rg2 and Rb1. It has a high medicinal value because of its anti-oxidant, anti-aging, and anti-tumor effects. There are some studies reporting the role of ginsenoside in prevention and treatment of DKD. For instance, ginsenoside Re played its therapeutic effect against DKD by reducing the levels of blood glucose and lipid and ameliorating oxidative stress [121]. In addition, ginsenoside Rb1 reversed the increase in expression of mitochondrial protein NADPH oxidase 4 (NOX4) to reduce the apoptosis and glomerular injury induced by CytoC and Caspase 9 [122]. Ginsenoside Rg1 was reported to have effects in reducing EMT in podocytes by enhancing the Akt/GSK3β pathway and restoring autophagy [123]. Additionally, it could also reduce inflammation, oxidative stress and apoptosis through promoting FoxO3 nuclear translocation *via* the PI3K/Akt pathway [124]. Combination of ginsenoside with astragaloside was conducive to improving renal function because of their effects in ameliorating oxidative stress and inhibiting the TGF-β1/Smad signaling [125]. Ginsenoside Rg5 was reported to decrease ROS generation to resist oxidative stress and inhibit the activation of NLRP3 inflammasome to ameliorate the renal tissue inflammation in diabetic mice [126].

Notoginsenoside could inhibit VEGF and TGF-β1 to increase BMP-7 and Smad7, lowering glucose and reducing FBG, CCr, urinary albumin (UAlb) and kidney index [127].

#### 3.1.6. Other

Emodin is a bioactive anthraquinone from Rhubarb. It was reported that emodin reduced the NF-κB-mediated expression of TGF-β1 and FN, thereby decreasing the high glucose-induced EMT [128]. Moreover, the *in vivo*/vitro experiments performed by Tian NX et al. [129] revealed that emodin suppressed the PERK-elf2α signaling to reduce ER stress, in turn to decrease podocyte apoptosis and improve renal function. Multiple studies also proved that emodin could promote the AMPK/mTOR-mediated autophagy [130] and active the PI3K/Akt/GSK-3β pathway to inhibit inflammation, apoptosis, and prevent DKD [131].

### 3.2. Chinese patent medicine

Chinese patent medicines are pharmacological agents prepared by single or multiple Chinese herbal medicines on the basis of TCM theory, and have a long history. Because of the long course and complicated mechanism of DKD, multiple Chinese patent medicines, such as Tangshen Formula and Wuling Powder, have achieved good efficacy in clinical practice owing to their multi-component and multi-target properties. See Table 2 for more details.

**Table 2.** Clinical efficacy and mechanism of action of Chinese patent medicine for DKD treatment.

Chinese patent medicine	Result	Pathway
Tangshen Formula	Reduce blood glucose and urinary albumin to resist inflammation and fibrosis	JAK/STAT/SOCS1, TNF-α and TGF-β/MMP-1 pathways, Col-IV level [133]
Huangkui Capsule	Prevent tubulointerstitial fibrosis, reduce SCr and BUN, improve inflammation	NADPH, ERK and TGF-β1/p38-MAPK pathways [135–137]
Yishen Tongluo Granule	Improve Ccr, FBG, TG, TC and HbA1c, etc.	Regulate expression of SOD, MDA, ET-1 and VEGF [138]
Six-Ingredient Rehmannia Pill	Reduce the UAER and β2-microglobulin in blood and urine	Inhibit ROS production [139–141]
Wuling Powder	Reduce blood glucose and improve glycation-mediated kidney injury	Suppress NF-κB and TGF- β /Smads pathways [142–143]
Jinshuibao Capsule	Reduce SBP, DBP, and 24-h UP, UACR, SCr, BUN and TG	Regulate glycolipid metabolism, anti-oxidization, and anti-inflammation [144–146]
Tongluo Capsule	Improve SCr, BUN, TC, TG, LDL-C, etc.	Decrease serum TGF-β level [147]
Shenshuaining Granule	Reduce 24-h UP, SCr and BUN	Inhibit SOD, AOPP, AGEs and IL-8 [148]
Compound Rhizoma Coptidis Capsule	Reduce FBG, BUN, SCr, and 24-h UP	Inhibit TGF-β1 and Col-IV [149]
Qizhi Jiangtang Capsule	Reduce urinary protein	Anti-oxidative stress [150]
Qi-flavor Granule	Anti-inflammation, and anti-oxidative stress	Decrease TGF-β1 expression [151]
Chaihuang Yishen Granule	Reduce 24-h UP	Inhibit the TGF-β/Smad3-mediated kidney fibrosis [152]
Qidan Dihuang Granule	Improve 24-h urinary proteinuria, total proteinuria, and UACR	Reduce fibrosis in glomerular endothelial and mesangial cells [153]

Tangshen Formula is prepared by Milkvetch Root, Unprocessed Rehmannia Root, Asiatic Cornelian Cherry Fruit, Sanqi, Winged Euonymus, Cooked Rhubarb and Orange Fruit and has functions of tonifying qi and yin and activating blood. In a clinical trial involving 144 DKD patients, 50 weeks of treatment with the combination of Tangshen Formula and Irbesartan (2-week lead-in period, 24-week intervention period, and 24-week follow-up period) led to a significant reduction in 24-h UP [132]. Scientific research is also conducive for effective clinical treatment. For instance, animal experiment in db/db mice revealed that the Tangshen Formula reduced blood glucose and UAlb *via* the JAK/STAT/SOCS1 signaling [133]. Moreover, the Tangshen Formula could also protect the kidney *via* its anti-inflammatory and anti-fibrotic effects by down-regulating TNF-α, inhibiting TGF-β, enhancing MMP-1 and decreasing Col-IV level.

Huangkui Capsule is prepared by the extracts of the flower of Abelmoschus manihot(L.), and it is the agent with various functions such as anti-oxidization, anti-inflammation and immunomodulation. It has been recommended for clinical treatment of DKD and chronic nephritis, etc. Evidence-based data proved that Huangkui Capsule had significant therapeutic efficacy for proteinuria and could effectively delay the deterioration of renal function [134]. Pharmacological studies demonstrated that Huangkui Capsule administration could inhibit NADPH oxidase activity, decrease ROS production, and block the ERK signaling [135] or suppress the TGF-β1/p38MAPK pathway [136] to prevent tubulointerstitial fibrosis. In addition, it could reduce SCr, BUN and activate PPAR to ameliorate ER stress, improving lipid metabolic disorders and alleviating inflammation [137]. Given the good therapeutic efficacy, Huangkui Capsule deserves clinical promotion.

Yishen Tongluo Granule, prepared by Salvia Root, Milkvetch Root, Asiatic Cornelian Cherry Fruit, Rhubarb, Prepared Rehmannia Root, Dragon’s Blood and Euonymus alatus, is one of the classical Chinese compound formulae used for DKD treatment. It mainly acts to tonify the kidney and dredge collaterals. Clinical research reported that Yishen Tongluo Granule was superior to Benazepril in improving renal function and decreasing the levels of endogenous CCr, FBG, HbA1c, TC and triglyceride (TG). It was believed that Yishen Tongluo Granule exhibited these effects *via* inhibiting SOD and MDA and regulating ET-1 and VEGF expression [138].

Six-Ingredient Rehmannia Pill, prepared by Prepared Rehmannia Root, Common Yam Rhizome, Asiatic Cornelian Cherry Fruit, Tree Peony Root Bark, Poria and Oriental Waterplantain Rhizome, has good clinical therapeutic efficacy for DKD. Hsu PC et al. [139] established a regression model for predicting the risk of developing renal failure in DKD patients with and without Six-Ingredient Rehmannia Pill, and they found that administration of Six-Ingredient Rehmannia Pill during diabetic care slowed down the occurrence of DKD. Another Cox proportional hazard regression model based on 70,036 diabetic patients revealed that Six-Ingredient Rehmannia Pill administration significantly delayed the use of hypoglycemic agents and insulin [140]. Additionally, experiments demonstrated that Six-Ingredient Rehmannia Pill exhibited significant nephroprotective effects *via* reducing glomerular mesangial matrix expansion and basement membrane thickening. In the meantime, it also suppressed the activity of AR in hemocytes and reduced the levels of UAER and β2-microglobulin in the blood and urine without affecting the blood glucose and lipid and mean arterial pressure (MAP), which might be associated with the inhibition of ROS production [141].

Wuling Powder is also known as Poria Five-herb Formula due to its five components including Poria, Oriental Waterplantain Rhizome, Chuling, White Atractylodes Rhizome and Cassia Twig. It is a representative formula for treatment of greater yang water retention syndrome and can ameliorate the edema in late-stage DKD. Clinical trial suggested that Wuling Powder used as an adjuvant of Rosiglitazone alleviated edema and improved lipid metabolism while increasing urinary volume and decreasing proteinuria, reaching a response rate of up to 94.1% (76.5% in control group) [142]. In addition, animal experiment also showed that Wuling Powder decreased the NF-κB and TGF-β1 ex pression in STZ-induced diabetic rats, which lowered blood glucose and improved the glycation-mediated kidney injury, showing a nephroprotective role [143].

Jinshuibao Capsule, mainly prepared by the artificially fermented cordyceps sinensis Cs-4 strain, has extensive applications in clinical prevention and treatment of diabetes complicated by kidney injury and other chronic kidney diseases. It has nephroprotective and hepatoprotective roles as well as functions of immunomodulation and anti-inflammation, which have been proven in clinical practice [144]. Fermented cordyceps sinens is contains multiple amino acids, vitamins and trace elements, which allow for improvement of the amino acid metabolism inside and outside cells and the extracellular mitochondrial respiratory function, conducive to accelerating cell damage repair [145]. A meta-analysis investigated 4,562 patients from 48 studies and found that the combination of Jinshuibao Capsule with ACEI/ARB had superior efficacy and safety to ACEI/ARB alone in reducing 24-h UP, UACR, SCr, BUN and TG, HbA1c, TNF-α, and CRP [146].

Beside the common Chinese patent medicines mentioned above, Tongluo Capsule was reported to be more effective in improving the levels of SCr, BUN, TC, TG, low-density lipoprotein cholesterol (LDL-C) and high-density lipoprotein (HDL) [147]. Shenshuaining Tablet could significantly decrease the 24-h urinary microalbumin, SCr, and BUN [148]. Compound Rhizoma Coptidis Capsule, prepared by Golden Thread, Kudzuvine Root, Sarrette and Loquat Leaf, could inhibit the expression of TGF-β1 and Col-IV to protect kidney function and slow down DKD progression [149]. Qizhi Jiangtang Capsule, composed of Milkvetch Root, Leech, Rehmannia Root and Crystalline Lens, could effectively reduce UP to delay the progression of renal disorder [150]. Qi-flavor Granule, comprising Milkvetch Root, Prepared Rehmannia Root, Winged Euonymus and Rhubarb, could ameliorate the kidney lesions and decrease TGF-β1 expression in type 2 DM KK-Ay mice [151]. Chaihuang Yishen Electuary is composed of Milkvetch Root, Chinese Yam, Chinese Thorowax Root, Chinese Angelica, Baical Skullcap Root, Chuling and Leech and could be employed as an agent for DKD treatment because of its effect to block the TGF-β/Smad3-mediated kidney fibrosis [152]. Qidan Dihuang Granule could profoundly improve the levels of 24-h urinary proteinuria, total proteinuria and 4-d UACR [153]. Danggui Shaoyao Powder, a combination of Debark Peony Root, Chinese Angelica, Sichuan Lovage Rhizome, Poria, White Atractylodes Rhizome and Oriental Waterplantain Rhizome, could protect kidney function in STZ-induced diabetic rats *via* regulating blood glucose and decreasing the expression of AGEs in diabetic glomerulus [154]. Xueshuangtong Compound Granule, composed of Sanqi, Salvia Root, Figwort Root and Milkvetch Root, could prevent glomerular hypertrophy and mesangial matrix expansion, and decrease the activity of SOD and MDA [155].

## 4. Summary and prospect

DKD is one of the important microvascular complications of diabetes, and its onset is closely associated with multiple mechanisms such as inflammation, blood lipid metabolic disorder, and hemodynamics abnormality. Notably, oxidative stress is the core of various mechanisms. In the present review, we summarized the upstream pathways that induce oxidative stress and the downstream pathways that are activated by oxidative stress, in an attempt to explore the role of oxidative stress in pathogenesis of DKD.

Upon sustained high glucose, multiple pathways such as AGEs, PKC and hexosamine pathways are activated, stimulating ROS production and triggering oxidative stress. As a result, the body’s oxidant-antioxidant balance is impaired, resulting in cascade reactions involved in multiple downstream pathways (e.g., P13K/AKT, TGF-β1/p38-MAPK, and NF-κB) that lead to inflammation, fibrosis and apoptosis, eventually exacerbating the progression of DKD. The body’s oxidant and antioxidant systems are complicated and interacted. ROS production induces oxidative stress, while the oxidative stress response in turn stimulates ROS accumulation *via* multiple pathways. These processes interact to form a vicious cycle, which can cause damage to podocytes and kidney tissue. In this context, enhancing the ability of the antioxidant defense system (such as increasing the activity of antioxidant enzymes) and blocking oxidative stress-related pathways might be a new direction for research and development of therapeutic agents for DKD.

Chinese medicine is a treasure of the Chinese nation. Either herbal medicines or their active ingredients or patent medicines take an active role in DKD prevention because of their multi-component and multi-target properties. The present review described the current advances in TCM intervention for DKD treatment, so as to provide evidence for further research and development of Chinese medicines applicable for clinical DKD treatment. It has been recognized that the protective role of Chinese medicines in DKD is largely attributed to their anti-oxidant, anti-inflammatory, anti-fibrotic, and podocyte-protective effects. Nonetheless, there are still some issues that need to be addressed. For example, the specific mechanism by which Chinese medicine prevents DKD *via* interfering with oxidative stress is still an open issue and lacks comprehensive studies. Presently, the research into some active ingredients of Chinese medicines or patent medicines is limited to cellular and animal experiments, without support from clinical trials. Besides, in spite of the certain clinical efficacy, further validation toward safety is in demand due to the small sample size. Therefore, it is necessary to carry out multi-center, multi-level, large-scale clinical trials to ensure the effectiveness, tolerance, and safety of Chinese medicines. Moreover, relevant signaling pathways that are involved in their mechanisms of action are not explored yet, requiring further comprehensive studies to help improve the clinical service. Research directions in the future may include, but are not limited to DNA methylation, histone modification, RNA editing, miRNA, siRNA, lncRNA, and their crosstalks with traditional pathways.

To conclude, oxidative stress is critical for DKD pathogenesis, while Chinese medicines can inhibit the occurrence of oxidative stress to enhance the body’s anti-oxidant ability thereby to delay disease progression.
